# Cognitive complaints in patients with untreated obstructive sleep apnea versus patients with neurological and respiratory diseases: prevalence, severity and risk factors

**DOI:** 10.1007/s11325-024-03056-7

**Published:** 2024-05-18

**Authors:** Tim Vaessen, Ruth E. Mark, Wouter De Baene, Karin Gehring, Sebastiaan Overeem, Margriet M. Sitskoorn

**Affiliations:** 1 Department of Psychiatry and Medical Psychology, Spaarne Gasthuis, Boerhaavelaan 22, 2035 RC Haarlem, the Netherlands; 2 Department of Cognitive Neuropsychology, Tilburg University, Tilburg, the Netherlands; 3 Department of Neurosurgery, Elisabeth-TweeSteden Hospital, Tilburg, the Netherlands; 4 Sleep Medicine Center “Kempenhaeghe”, Heeze, the Netherlands; 5 Department of Electrical Engineering, Eindhoven University of Technology, Eindhoven, the Netherlands

**Keywords:** Brain tumor, Cognitive complaints, Cognitive functioning, Cognitive impairments, Obstructive sleep apnea, Pulmonary diseases, Stroke

## Abstract

**Purpose:**

Little is known about cognitive complaints (self-reported problems in cognitive functioning) in patients with Obstructive Sleep Apnea (OSA). We compared the prevalence and severity of cognitive complaints in patients with untreated OSA to patients with neurological and respiratory diseases. We also studied risk factors for cognitive complaints across these diseases, including OSA.

**Methods:**

We used a convenience sample to compare untreated OSA patients (*N* = 86) to patients with stroke (*N* = 166), primary brain tumor (*N* = 197) and chronic obstructive pulmonary disease (COPD, *N* = 204) on cognitive complaints (Cognitive Failure Questionnaire, CFQ), anxiety and depression (Hospital Anxiety and Depression Scale, HADS) and cognitive impairments using neuropsychological tests. We combined all patient groups (OSA, stroke, brain tumor and COPD) and studied potential risk factors (demographic variables, anxiety, depression and cognitive impairments) for cognitive complaints across all patient groups using regression analysis.

**Results:**

The prevalence of cognitive complaints was higher in OSA patients and complaints of forgetfulness and distractibility were more severe compared to stroke and primary brain tumor patients, but similar to or lower than COPD patients. Regression analysis for the combined sample of all patient groups showed that cognitive complaints were most strongly associated with symptoms of anxiety and depression.

**Conclusion:**

A high rate of OSA reported clinically significant cognitive complaints, comparable to other respiratory and neurological patients. Symptoms of anxiety and depression are important risk factors for cognitive complaints in patients with various neurological and respiratory diseases. Future studies should examine the relation between anxiety, depression and cognitive complaints in patients with OSA.

## Introduction

Obstructive Sleep Apnea (OSA) is a sleep disorder characterized by intermittent obstruction of the airway during sleep. OSA is associated with cognitive impairments as verified with neuropsychological testing [1]. Causes for cognitive impairments in OSA are multifactorial and include disruption of sleep and nighttime blood gas abnormalities (hypoxemia). More recent studies show that fragmented sleep and hypoxemia may be insufficient to cause cognitive impairments [2–4]. These studies show that comorbid conditions, such as overweight, hypertension, stroke, diabetes and emotional problems may worsen or even be the primary cause of cognitive impairments [5, 6]. Impairments in cognition in OSA might therefor be similar to or have overlap with those other medical diseases [5]. OSA is often treated with continuous positive airway pressure (CPAP). While CPAP improves cognition, residual cognitive impairments may persist post-treatment [6, 7].

Fundamentally different from objectively tested cognitive impairments are cognitive complaints. These are subjective self-reported problems in cognitive functioning (with or without objective cognitive impairment). Cognitive complaints are concerns patients express about their daily life cognitive functioning and are an important reason patients seek medical help for their cognition. They can be assessed relatively easily through an interview or by validated questionnaires. Evaluating cognitive complaints alongside objective cognitive impairments is crucial, as they correlate to overall well-being [8], other self-reported outcomes [9, 10], and absence from work due to sickness [11].

Cognitive complaints are common in patients with neurological diseases, such as stroke [12], and brain tumors [10], and respiratory diseases, such as chronic obstructive pulmonary disease (COPD) [13]. The limited studies in untreated OSA indicate more severe concentration problems compared to healthy controls [14]. To our best knowledge, no study has compared the prevalence and severity of cognitive complaints in patients with untreated OSA to other patients with neurological or respiratory diseases.

Associated with cognitive complaints include/are symptoms of anxiety and depression [15], objective cognitive impairments [9], and a chronic (versus acute) phase of the disorder [16]. Here, the acute phase typically refers to the early onset of symptoms (days to weeks) whereas the chronic phase refers to the months or years in which symptoms remain relatively stable. Given the chronic nature of OSA, and that it is often accompanied by depression, anxiety symptoms [17], and objective cognitive impairments [18], it is expected that cognitive complaints in OSA may be as prevalent and severe as those in patients with other diseases.

To test this hypothesis, we performed a retrospective study using available datasets on cognitive complaints in patients with OSA, stroke, primary brain tumors and COPD. We are aware that some of these medical diseases share comorbidities, such as hypertension or even OSA. However, our purpose is not to determine the unique contribution of OSA on cognitive complaints in various diseases but to compare cognitive complaints between untreated OSA patients and patients with other neurological and respiratory diseases, regardless of underlying pathophysiological causes of cognitive complaints or shared comorbidities. Additionally, we studied potential risk factors (sociodemographic characteristics, anxiety and depression symptoms and cognitive impairments) for cognitive complaints across all four patient groups.

## Methods

### Participants

OSA patients were recruited from the sleep units of two general hospitals in the Netherlands (VieCuri Medical Center, Venlo; and Reinier de Graaf Hospital, Delft). OSA diagnosis followed the clinical guidelines of the adult OSA taskforce of the American Academy of Sleep Medicine: apnea/hypopnea-index (AHI) of ≥ 15 or AHI of ≥ 5 according to a polysomnography (PSG) with significant daytime symptoms [19]. Patients were assessed before they started treatment.

We compared OSA patients (*N* = 86) to three separate medical diseases (these three groups were previously recruited for other studies). These were patients three months after a stroke without major communication difficulties (*N* = 166) [20] and patients three months after surgery for a primary brain tumor: low grade glioma (*N* = 85) [21], or meningioma (*N* = 112) [9]. All neurological patients were recruited at the general hospital where they received their treatment. Our third group comprised patients suffering from COPD (*N* = 204) [22]. They were recruited at a tertiary center for pulmonary rehabilitation. The majority of these patients had severe to very severe airway obstruction global initiative for chronic obstructive lung disease stage of III–IV based on pulmonary function parameter [23]. Exclusion criteria for OSA, stroke, COPD and brain tumor patients were a history of a psychiatric or (other) neurological disorder and the use of medication/substances impacting cognition. All studies were conducted in accordance with the declaration of Helsinki and approved by nationally certified medical ethical committees (OSA: NL37795.068.11; stroke: NL21642.008.08; low grade glioma + meningioma: NL41351.008.12; COPD: NL33713.008.10).

### Measures

#### Sociodemographic characteristics

Data on age, sex and educational level were collected for all patient groups. Educational level was rated according to the Dutch Verhage system: a 7-point scale similar to the International Standard Classification of Education [24], but adapted to the Dutch educational system.

#### Cognitive complaints

Cognitive complaints were assessed using the Cognitive Failure Questionnaire (CFQ) [25]. The CFQ is a validated and reliable instrument that assesses the severity of everyday cognitive complaints in the last three months and consists of 25 items. Every item is rated on a 5-point Likert-scale (ranging from “never” to “very often”). Higher scores indicate a higher severity of everyday cognitive complaints. Scores were classified as clinically significant if they were one standard deviation above the mean for a healthy Dutch norm sample (> 68) [26].

**Fig. 1:**
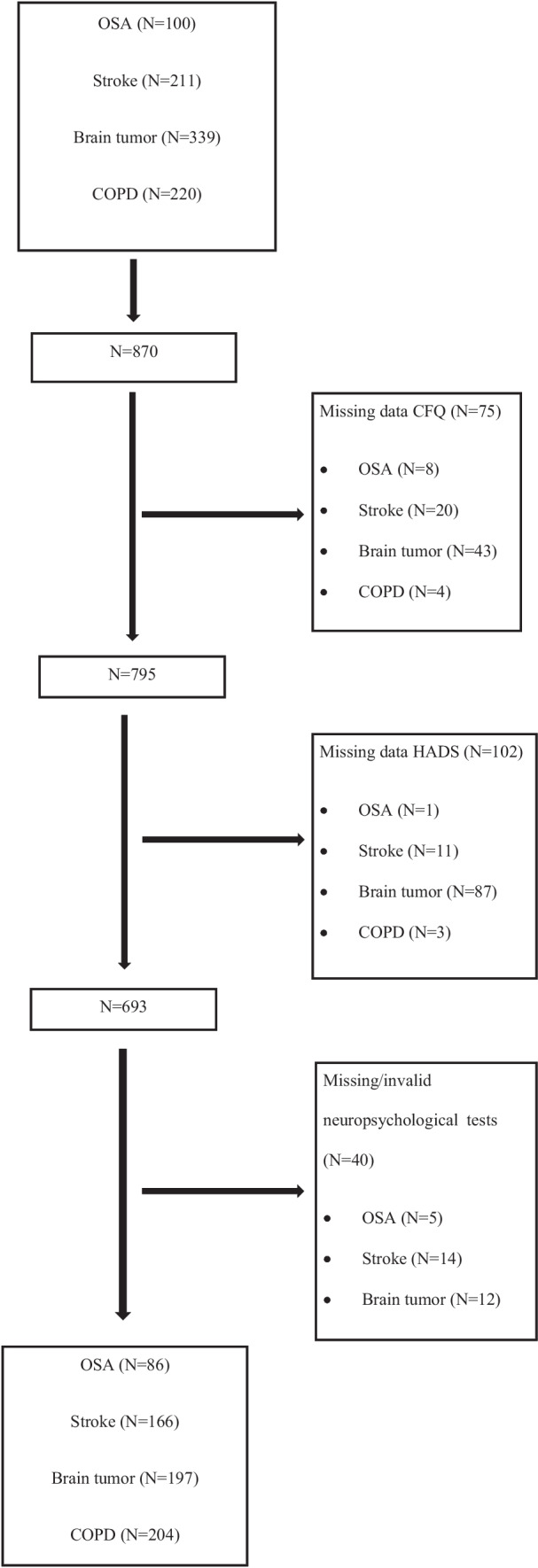
Flow chart of inclusion

Rast and colleagues found a stable factor structure of the CFQ across different ages in a Dutch population consisting of three subscales: “forgetfulness” (sum of 8 items, score range 0–32), “distractibility” (sum of 8 items, score range 0–32) and “false-triggering” (sum of 8 items, score range 0–32) [27]. The subscale forgetfulness contains items related to memory slips. The subscale distractibility contains items related to attentional misses, such as absentmindedness. The subscale false-triggering contains items related to blunders or slips in thinking or motor actions. If one item was missing on any of the CFQ subscales it was replaced by the average score of the same subscale. If more than one item was missing on any of the CFQ subscales that subscale was labeled as missing (see Fig. 1).

#### Depression and anxiety symptoms

Depression and anxiety symptoms were measured using the depression (sum of 7 items, score range 0–21) and anxiety subscales (sum of 7 items, score range 0–21) of the Hospital Anxiety and Depression Scale (HADS), a reliable and validated questionnaire for mood problems in a medical setting [28]. If one item was missing on any of the HADS subscales the average score of the subscale replaced it. If more than one item was missing on any of the two subscales, the subscale was labeled as missing (see Fig. 1). A score of 8 or higher on either of the subscales indicates clinical symptoms of depression or anxiety.

#### Cognitive impairments

Neuropsychological tests were used to assess cognitive performance. We defined cognitive impairments using the Cognitive Impairment Non-Dementia (CIND) criteria [29]. These criteria were selected because previous studies have shown CIND criteria to be useful in defining cognitive impairments in various medical diseases [30]. According to these criteria, a cognitive domain is impaired if the norm-corrected Z-score is below -1.65 after adjusting for age, sex and educational level (the lowest 5% of the normal population). For brain tumor patients, scores were also corrected for practice effects [31], as this group had already taken the same tests three months prior. See Table 1 for all cognitive tests and scores per domain used for the different patient groups.

**Table 1:** Cognitive tests and scores used per cognitive domain for all patientgroups

Cognitive domain	Test	Scores used*	Patientgroup
Verbal memory	RAVLT-DR [49]	Total number of correct responses on delayed recall	OSA
	WMS-PA-DR [35]	Total number of correct responses on delayed recall	Stroke
	CNSVS-VM [50]	Total number of correct responses on delayed recall	Brain tumor, COPD
Cognitive flexibility	CNSVS-CF [50]	Total number of correct responses and errors on the Shifting Attention Test and the number of commission errors of the Stroop test	OSA, brain tumor, COPD
	SCWT [36]	Interference score: based on the time to complete the interference part of the SCWT corrected for an estimated time based on the naming part of the SCWT test	Stroke
Processing speed	CNSVS-PS [50]	Number of correct responses minus the number of errors on the Symbol Digit Coding test (an adaptation of the pen-and-paper Digit Symbol Substitution Test)	OSA, brain tumor, COPD
	WAIS-SDST [35]	number of correctly coded symbols	Stroke

*All scores were corrected for age, sex and educational level using available Dutch norm groups, except for WMS-PA-DR and WAIS-SDST, for these scores only age corrected norm scores were available

CNSVS-CF = Central Nervous System Vital Signs Cognitive Flexibility Domain, CNSVS-PS = Central Nervous System Vital Signs Processing Speed Domain, CNSVS-VM = Central Nervous System Vital Signs Verbal Memory Domain, RAVLT-DR = Rey Auditory Verbal Leaning Test Delayed Recall, SCWT = Stroop Color Word Test, WAIS-SD = Wechsler Adult intelligence Scale III Symbol Digit Substitution Test, WMS-PA-DR = Wechsler Memory Scale III Paired Association Delayed Recall

##### Verbal memory

In OSA patients, verbal memory was measured using the delayed recall score (total number of correct responses) of the Dutch version of the Rey Auditory Verbal Learning Test (RAVLT-DR) [32]. For COPD and brain tumor patients, the Verbal Memory domain score of Central Nervous System Vital Signs (CNSVS-VM) was used [33]. The CNSVS is a computerized neurocognitive test battery developed as a routine clinical screening instrument. This verbal memory test is based on an adaptation of the Rey Auditory Verbal Learning Test [34] and is recognition-based. Correct responses are summed to generate the composite Verbal Memory domain score. In stroke patients, the delayed recall score (total number of correct responses) of the Verbal Paired Association subtest of the Wechsler Memory Test (WMS-PA-DR) third edition [35] was used. For WMS-PA-DR only age- (not sex and educational level) corrected norms were available.

##### Cognitive flexibility

In OSA, COPD and brain tumor patients, cognitive flexibility was measured using the Cognitive Flexibility domain of Central Nervous System Vital Signs (CNSVS-CF) [31]. The score on this domain is based on the total number of correct responses and errors on the Shifting Attention Test (a measure of cognitive flexibility) and the number of commission errors of the Stroop test (a computerized version of the pen-and-paper Stroop Color Word Test, SCWT). In stroke patients, the interference score of the pen-and-paper SCWT was used as a measure for cognitive flexibility [36]. The SCWT consists of a reading, naming and interference part. The interference score was based on the time to complete the interference part corrected for an estimated time based on the naming part of the test [37].

##### Processing speed

In OSA, COPD and brain tumor patients processing speed was measured using the Processing Speed domain of Central Nervous System Vital Signs (CNSVS-PS) [31]. The score of this domain is based on the number of correct responses minus the number of errors on the Symbol Digit Coding test (an adaptation of the pen-and-paper Digit Symbol Substitution Test). In stroke patients, processing speed was measured using the pen-and-paper Digit Symbol Substitution Test of the Wechsler Adult Intelligence Scale (WAIS-DSST) third edition [35]. The score is based on the number of correctly coded symbols. For WAIS-DSST only age- (not sex and educational level) corrected norms were available.

### Statistical analysis

Patients with untreated OSA were compared to patients with stroke, brain tumor and COPD on sociodemographic variables (age, sex and educational level), prevalence of cognitive complaints (percentage of patients with significant elevated total CFQ scores), severity of cognitive complaints (scores on CFQ subscales for forgetfulness, distractibility and false triggering), anxiety and depression symptoms (scores on HADS) and cognitive impairments (impaired scores on tests for verbal memory, cognitive flexibility and processing speed). For continuous variables we used independent samples t-tests and for categorical variables Chi-square tests.

We combined all patient groups and performed separate multiple linear regression analyses for the three CFQ subscales. As predictors we used age, sex, educational level, HADS depression, HADS anxiety and cognitive impairments. Standardized regression coefficients (β) were used to indicate the strength of the associations. We only examined component measures if the overall multivariate effect was significant. Two-sided p-values are reported and a p-value < 0.05 was considered to indicate statistical significance. P-values were adjusted using false discovery rates with the Benjamini–Hochberg method to correct for multiple testing [38]. All statistical analyses were performed using SPSS 24.0.0.0 software for Windows (IBM Corp, 2017).

## Results

### Participants

Figure 1 shows the inclusion flow chart for all included patient groups. We included a total of 870 patients of whom 170 had missing questionnaires cores (CFQ and/or HADS) and 40 had missing or invalid scores on the neuropsychological tests. In our analyses we were therefor able to include 86 OSA, 166 stroke, 197 brain tumor and 204 COPD patients.

### Patient and sociodemographic characteristics

Table 2 shows the patient characteristics of the four patient groups. OSA severity ranged from mild to severe (AHI range 5.2—101.2) with a median of 16.2 and interquartile range of 21.5. OSA patients were significantly more often male than any of the other patients (all p’s < 0.04). OSA patients were significantly younger than stroke and COPD patients (all p’s < 0.001), but did not differ in age from brain tumor patients (*p* = 0.21). Furthermore, OSA patients had, on average, a significantly higher educational level than stroke (*p* = 0.01) and COPD patients (*p* < 0.001) while they did not differ on this variable from the brain tumor patients (*p* = 0.24).

**Table 2:** Demographic variables, cognitive complaints, depression, anxiety and cognitive impairments

	Range	OSA	Stroke	*p*-value^1^	Brain tumor	*p*-value^1^	COPD	*p*-value^1^
		*N* = 86	*N* = 166		*N* = 197		*N* = 204	
Demographics								
Sex (% female)	0–100	21%	34%*	.04	66%***	< .001	52%	< .001
Age	> 18	50.0 (10.2)	64.1 (12.6)***	< .001	52.3 (13.3)	.21	61.3 (8.6)	< .001
Education	1–7	5.3 (1.0)	4.6 (1.4)*	.01	5.0 (1.2)	.24	4.1 (1.2)	< .001
AHI	> 5	24.5 (19.7)	na	na	na	na	na	
Cognitive complaints								
CFQ significant cognitive complaints (%)	0–100	30.2%	17.5%*	.04	13.8%**	< .01	22.7%	.22
CFQ forgetfulness	0–32	13.3 (5.1)	11.4 (5.2)*	.01	11.1 (5.7)**	< .01	13.2 (4.4)	.83
CFQ distractibility	0–32	11.6 (4.8)	9.4 (5.0)**	< .01	8.9 (5.1)***	< .001	11.8 (4.0)	.84
CFQ false triggering	0–32	8.3 (4.9)	6.8 (4.4)*	.03	6.8 (4.6)*	.03	8.9 (4.1)	.34
Depression and anxiety								
HADS depression score	0–21	5.3 (3.5)	5.2 (3.8)	.80	3.6 (3.1)***	< .001	7.1 (3.9)	< .001
HADS clinical symptoms depression(%)	0–100	29.1%	30.1%	.86	9.6%***	< .001	44,1%	.03
HADS anxiety score	0–21	5.9 (3.3)	4.7 (3.9)*	.04	4.6 (3.7)*	.02	7.3 (4.3)	.01
HADS clinical symptoms anxiety (%)	0–100	29.1%	21.1%	.21	18.8%	.08	50.0%	< .01
Cognitive impairments								
Verbal memory (%)	0–100	24.0%	8.7%*	.01	26.9%	.57	14.5%	.09
Cognitive flexibility (%)	0–100	30.2%	2.4%***	< .001	23.4%	.14	25.4%	.23
Processing speed (%)	0–100	8.2%	19.3%	.06	17.8%	.10	26.4%	.01

AHI = Apnea/Hypopnea Index, CFQ = Cognitive Failure Questionnaire, HADS = Hospital Anxiety Depression Scale, na = not applicable,

^1^compared to OSA, * < .05, ** < .01, *** < .001

### Cognitive complaints

In OSA 30.2% reported significant cognitive complaints (total CFQ score of more than one standard deviation above the mean of Dutch norms (see Table 2)). This percentage was significantly higher than in patients with stroke (*p* = 0.04) and with a brain tumor (p < 0.01), but did not differ compared to COPD patients (*p* = 0.22). OSA patients also reported a higher mean on the CFQ subscales for forgetfulness, distractibility and false triggering than stroke and brain tumor patients did (all p’s < 0.03, see Table 2). OSA and COPD patients did not differ in mean scores on any of the CFQ-subscales (all p’s > 0.34).

### Depression and anxiety symptoms

In OSA patients, 29.1% reported clinical symptoms of depression (HADS depression) and 29.1% reported clinical symptoms of anxiety (HADS anxiety). These numbers were significantly lower compared to COPD patients for both clinical symptoms of depression and anxiety (all p’s < 0.03). OSA patients reported a higher number of clinical symptoms of depression compared to brain tumor patients (*p* < 0.001). For clinical symptoms of anxiety no differences were found between OSA and brain tumor patients (*p* = 0.08). No differences were also found between OSA and stroke for the number of patients with clinical symptoms of depression and anxiety (all p’s > 0.21).

OSA patients had a significantly higher overall score on HADS depression and anxiety than brain tumor patients (all p’s < 0.05), but a significantly lower overall score on HADS depression and anxiety than COPD patients (all p’s < 0.05). Compared to stroke patients, OSA patients scored significantly higher on HADS-anxiety (*p* < 0.05), but there was no difference on HADS-depression (*p* = 0.80).

### Cognitive impairments

In OSA patients, 24% had an impairment in verbal memory, 30% in cognitive flexibility and 8% in processing speed (see Table 2). A significantly higher proportion of OSA patients had an impairment in verbal memory and cognitive flexibility (all p’s < 0.05) compared to stroke patients, but there was no significant difference for processing speed (*p* = 0.06). A significantly lower number of OSA patients had a cognitive impairment in processing speed compared to COPD (*p* < 0.05), with no significant differences for verbal memory and cognitive flexibility (all p’s > 0.09). OSA patients also did not significantly differ in the proportions of cognitive impairments on any domain compared to brain tumor patients (all p’s > 0.10).

### Risk factors for cognitive complaints

For all three subscales of the CFQ we performed separate multivariate regression analyses (see Table 3). We entered age, sex, education, HADS depression score, HADS anxiety score and cognitive impairments in verbal memory, cognitive flexibility and processing speed as candidate predictors. All three models were significant (CFQ forgetfulness: F (8,651) = 13.5, *p* < 0.001, CFQ distractibility: F (8,651) = 19.6, *p* < 0.001, CFQ false triggering: F (8,651) = 14.5, *p* < 0.001), with R^2^’s of 0.14 for CFQ forgetfulness, 0.20 for CFQ distractibility and .15 for CFQ false triggering.

**Table 3:** Multilinear regression analyses for all three CFQ subscales in the total sample

	CFQ forgetfulness N = 652		CFQ distractibility N = 652		CFQ false triggering N = 652	
Variables	Standardized ß	p-value	Standardized ß	p-value	Standardized ß	p-value
Sex	.06	.21	-.09*	< .05	.12**	.001
Age	.00	.96	-.09*	< .05	.00	.96
Education	.04	.42	-.08	.10	-.01	.83
HADS depression score	.25***	< .001	.20***	< .001	.18***	< .001
HADS anxiety score	.16**	.001	.26***	< .001	.21***	< .001
Verbal memory impairment	.07	.13	.06	.21	.08	.09
Cognitive flexibility impairment	-.06	.21	-.05	.28	-.03	.46
Processing speed impairment	-.04	.42	-.09*	< .05	-.05	.28

CFQ = Cognitive Failure Questionnaire, HADS = Hospital Anxiety and Depression Scale, OSA = Obstructive Sleep Apnea,

* < .05, ** < .01, *** < .001

For our combined sample of all patient groups we found that a higher score on CFQ forgetfulness (i.e. more severe complaints of forgetfulness) was associated with a higher score on HADS depression (ß = 0.25) and a higher score on HADS anxiety (ß = 0.16).

A higher score on CFQ distractibility (i.e. more severe complaints of distractibility) was associated with a higher score on HADS depression (ß = 0.20) and HADS anxiety (ß = 0.26). A lower score on CFQ distractibility (i.e. less severe complaints of distractibility) was associated with female sex (ß = -0.09), a younger age (ß = -0.09) and an impairment in processing speed (ß = -0.09).

A higher score on CFQ false triggering (i.e. more severe complaints of false triggering) was associated with a higher score on HADS depression (ß = 0.18), a higher score on HADS anxiety (ß = 0.21) and female sex (ß = 0.12),

## Discussion

To our knowledge, this is the first study comparing cognitive complaints in patients with untreated OSA to such complaints of patients with neurological or respiratory diseases. A high rate of our OSA sample reported clinically significant cognitive complaints (30.2%). Previous studies have shown that cognitive complaints are more prevalent in OSA compared to healthy controls [39, 40]. Our prevalence numbers are somewhat lower than what has been reported in previous studies (59% for memory complaints [40] and 69% for concentration complaints [41]). However, these studies used a single yes/no question to assess cognitive complaints. We do not know the percentages with similar reports of memory or concentration complaints in the healthy population. Because we used a validated questionnaire with norm-referenced cut-off scores, our prevalence numbers only include clinically significant cognitive complaints, not just any cognitive complaint also prevalent in the general population. This may explain why previous studies have found a higher prevalence of cognitive complaints in OSA.

The prevalence and severity of cognitive complaints in OSA were at least comparable to our sample of patients with other neurological and respiratory diseases. Cognitive complaints in OSA were even more prevalent and severe compared to our sample of neurological patients (stroke, brain tumor). We did not expect this last finding. There are four possible explanations for this. Firstly, patients with communication difficulties were excluded from the stroke group, likely excluding stroke patients with more severe cognitive complaints. Secondly, the datasets we used for stroke and brain tumors assessed patients three months after an acute neurological event (stroke, brain surgery). Patients at this stage of their disease may have not yet been confronted enough with cognitive failures to adequately report them [20]. Thirdly, after an acute medical event (such as stroke or brain tumor) patients may shift their evaluation of their health standards. Gratitude about their health may overshadow cognitive complaints [42]. Fourtly, we included OSA patients in the diagnostic phase at a sleep center. This sample probably has a bigger focus on and incentive to report cognitive complaints, compared to OSA patients that did not seek for help at a sleep center. Therefor the finding that OSA patients report more cognitive complaints than neurological patients need to be interpreted with caution. Current findings can only be generalized to OSA patients attending a sleep clinic and the prevalence and severity of cognitive complaints in our neurological sample might be an underestimation compared to the overall population of stroke and brain tumor patients.

For the combined sample (OSA, stroke, brain tumor and COPD together) cognitive complaints were most strongly associated with symptoms of depression and anxiety. This corresponds to results of previous cross-sectional studies in stroke [15], glioma [10], meningioma [9] and various other diseases [43]. Because our data is also cross-sectional the nature of this relation is unclear: cognitive complaints may be a sign of anxiety or depression, cognitive complaints may cause anxiety of depression or an unknown disease factor (such as fatigue or pain) may cause all three. Studies do show, however, that treating a depressive disorder reduces cognitive complaints [44]. This, together with our findings, implies that patients with cognitive complaints need to be screened for anxiety and depression. And if patients fit the criteria of a depressive disorder treatment for it will likely improve cognitive complaints.

A limitation of our study is that the medical diseases we included may share comorbidities, and there may be overlap between patient groups in the possible causes for cognitive complaints. For instance, OSA is also prevalent in stroke [45] and COPD [46] and OSA increases the risk for a primary brain tumor [47]. All included patient groups share comorbidities such as hypertension and diabetes mellitus. Unfortunately, the stroke, COPD and brain tumor patients we included were not screened for OSA. Also, data on comorbidities such as hypertension or diabetes mellitus was not available for all patient groups. However, for all patient groups we did exclude patients with psychiatric disorders, (other) neurological disorders and use of medication or substances that impact cognition. Therefore, we believe that the included patient groups are a good representation of patients in clinical practice with common comorbidities, but excluding comorbidities that have a large impact on cognition.

We were able to compare cognitive complaints in patients with untreated OSA to patients with other diseases using a well-validated questionnaire (CFQ). We also used well-established instruments to assess cognitive impairments. Almost all test scores were adjusted using age, sex and education corrected norms. This was done so we could compare across diseases, who inherently differ in age, sex and educational level. Although neuropsychological tests were often comparable across patient groups (e.g. a computerized adaptation versus a paper-and-pencil version of a test), it was not possible to use exactly the same tests (and norm scores) for all patient groups. We should therefore be careful when making comparisons between OSA and the other patient groups on cognitive impairments. This is most notable for stroke patients. For stroke patients a relatively easier and less sensitive verbal memory test was used compared to the patients with untreated OSA (WMS-PA-DR for stroke versus RAVLT-DR for OSA). WMS-PA-DR is less likely to pick up a verbal memory impairment than RAVLT-DR [48].

## Conclusions

Cognitive complaints are as prevalent and severe in patients with OSA as in patients with neurological diseases (stroke, primary brain tumors) and respiratory diseases (COPD). For all patient groups combined (OSA, stroke, brain tumor and COPD) symptoms of anxiety and depression were important risk factors for cognitive complaints. Our results underscore the importance of screening for symptoms of anxiety and depression in patients who express cognitive complaints. Future studies in OSA should examine the relation between cognitive complaints, anxiety, depression and cognitive impairments to assess which OSA patients are at risk for cognitive complaints.

## Data Availability

This study is based on the combined analysis of previously reported studies. With respect to data availability, we refer to the publications on the respective datasets. For the datasets of OSA the informed consent at the time of data collection does not allow data to be made available outside.

## Declarations

All authors have seen and approved this manuscript.

## Abbreviations

AHI: Apnea/Hypopnea Index
CNSVS: Central Nervous System Vital Signs
CNSVS-CF: Central Nervous System Vital Signs Cognitive Flexibility
CNSVS-PS: Central Nervous System Vital Signs Processing Speed
CNSVS-VM: Central Nervous System Vital Signs Verbal Memory
CFQ: Cognitive Failure Questionnaire
COPD: Chronic Obstructive Pulmonary Disease
CPAP: Continuous Positive Airway Pressure
ESS: Epworth Sleepiness Scale
HADS: Hospital Anxiety Depression Scale
OSA: Obstructive Sleep Apnea
RAVLT-DR: Rey Auditory Verbal Learning Test Delayed Recall
SCWT: Stroop Color Word Test
WAIS-DSST: Wechsler Adult Intelligence Scale Digit Symbol Substitution Test
WMS-PA-DR: Wechsler Memory Test Verbal Paired Association Delayed Recall
